# Weight Management Strategies to Reduce Metabolic Morbidity in Women With Polycystic Ovary Syndrome

**DOI:** 10.1007/s13679-025-00614-2

**Published:** 2025-03-06

**Authors:** Michail Diakosavvas, Oyinlola Oyebode, Priya Bhide

**Affiliations:** 1 Homerton Fertility Centre, Homerton Healthcare NHS Foundation Trust, London, UK; 2 Centre for Public Health and Policy, Wolfson Institute of Population Health, Queen Mary University of London, London, UK; 3 Yvonne Carter Building, 58 Turner Street, London, E12AB UK

**Keywords:** Polycystic ovary syndrome (PCOS), Obesity, Metabolic risk, Intensive weight management programmes (IWMPs), Glucagon-like peptide-1 receptor agonists (GLP-1 RAs), Obesity management, Insulin resistance

## Abstract

**Purpose of Review:**

Polycystic Ovary Syndrome (PCOS) affects 10–15% of women of reproductive age and is associated with a heightened risk of metabolic morbidity, exacerbated by insulin resistance and obesity. Current weight management strategies have limited effectiveness in reducing metabolic morbidity in this subgroup. This review examines the potential of Intensive Weight Management Programmes (IWMPs) and Glucagon-like peptide-1 receptor agonists (GLP-1 RAs) to reduce metabolic risks in women with PCOS, drawing from studies in both PCOS-specific and related populations.

**Recent Findings:**

IWMPs, including total diet replacement, achieve substantial and sustained weight loss (5–15% over 1–5 years) in individuals with obesity and type 2 diabetes, alongside improvements in metabolic markers like blood pressure and glycemic control. GLP-1 RAs, particularly semaglutide, similarly deliver significant weight loss (10–15% over 1–2 years) and metabolic benefits. While there is limited data specifically targeting PCOS, emerging studies suggest GLP-1 RAs can improve weight, insulin sensitivity, and menstrual regularity in this group. However, evidence for both interventions in PCOS remains insufficient.

**Summary:**

Women with PCOS face unique metabolic challenges, including heightened insulin resistance, compounded by obesity. While IWMPs and GLP-1 RAs are promising interventions, evidence for their effectiveness in PCOS-specific populations is insufficient. Addressing this research gap through targeted trials is essential to improve outcomes in individuals affected by PCOS and metabolic disorders.

## Introduction

Polycystic Ovary Syndrome (PCOS) is a common condition affecting 10–15% of women of reproductive age [1]. Women with PCOS have a significantly higher risk of developing metabolic morbidity [2]. The major factors contributing to the increased risk are inter-linked, and include insulin resistance, high levels of androgens and obesity [3]. The presence of metabolic morbidity significantly increases the risk of all-cause mortality, irrespective of obesity status [4]. Besides impacting individual health and quality of life, the management of metabolic morbidity has a significant economic impact for the health and social care service and a wider economic impact on society.

Insulin sensitivity and body weight are two key modifiable risk factors for metabolic morbidity in women with PCOS [1, 5, 6]. Multiple interventions have proven efficacy in addressing these factors, forming the basis for both UK and international clinical guidelines and recommendations [7–10]. These interventions include lifestyle advice (dietary changes and exercise) behavioural treatment, insulin sensitisers such as metformin, anti-obesity medications, anti-androgens, and bariatric surgery. The interventions are delivered at various tiers of the health service and the choice of intervention depends on body mass index (BMI) and the presence of co-morbidities. Similar guidelines and practices are recommended internationally, reflecting a global consensus on managing PCOS and its associated risks [1, 11, 12].

Metabolic morbidity in this subgroup of women however continues to be significant, bringing into question the effectiveness of the interventions in routine clinical care. This may be attributed to several factors. Although women with PCOS are inherently at a higher risk of developing metabolic morbidity by virtue of this diagnosis, PCOS is not explicitly identified as a co-morbidity in risk scoring systems such as the Qrisk score used by the National Health Service (NHS) in the UK. Hence most women with PCOS are treated with less intensive interventions, and specialist weight management services are either never initiated or initiated at a much higher body mass index (BMI) or after the development of co-morbidities. This disadvantages them as they may benefit from early initiation of more intensive treatment. Women from ethnic minority backgrounds, where PCOS is more common, experience type 2 diabetes (T2D) and cardiovascular disease (CVD) at a lower BMI due to excess abdominal fat [13, 14]. There is also significant heterogeneity in the interventions offered by healthcare professionals. This may be due to lack of definitive high-quality evidence for their long-term effectiveness, lack of funding and infrastructure for their implementation, disjointed clinical care pathways and inadequate uptake due to a variety of reasons [15–17]. Thus, an optimal weight management strategy to reduce metabolic morbidity in the specific subgroup of women with PCOS is lacking.

Intensive weight management programmes (IWMP) and Glucagon-like peptide 1 receptor agonists (GLP-1 RAs) are two effective intensive weight management strategies delivered in specialist weight management clinics. These are less commonly used in women with PCOS except at very high BMI and in the presence of co-morbidities. They may be effective for weight loss and reducing metabolic morbidity in women with PCOS at lower thresholds. IWMPs incorporate total diet replacement with structured food reintroduction and GLP-1 RAs work by appetite suppression, slower gastric emptying, and effects on glucagon and insulin metabolism.

Our objective was to evaluate the effects of IWMPs and GLP-1 RAs on weight loss and markers of metabolic morbidity in women with PCOS combining results from published literature.

## Methods

We employed a comprehensive search methodology that aimed to capture all relevant evidence pertaining to IWMPs and the anti-obesity medication GLP-1RA in populations with PCOS and metabolic diseases utilising the Medline and EMBASE bibliographic databases. We utilised MeSH and free text terms relating to the population and interventions studied. All types of studies published as primary research were included for the review. We included only those studies published in the English language, published as full manuscripts (not abstracts) and those involving humans-only. Recognizing the scarcity of studies specifically targeting PCOS populations in the initial search, we expanded our search to include individuals without PCOS but with similar metabolic morbidities. This broader approach enabled us to explore the implementation and effectiveness of these interventions across diverse populations with a primary focus on assessing metabolic morbidity outcomes.

## Results

### IWMPs

There were no studies identified that tested IWMPs in women with PCOS explicitly. For this reason, here we will examine the impact of IWMPs on populations with similar metabolic morbidities, that may have included women with PCOS.

The Diabetes Remission Clinical Trial (DiRECT) provided robust evidence supporting the efficacy of IWMPs in populations with type 2 diabetes (T2D). This cluster-randomized trial utilised the Counterweight-Plus program, incorporating an initial total diet replacement (TDR) phase followed by structured food reintroduction and ongoing weight maintenance. The trial demonstrated a substantial weight loss with an average absolute weight reduction of 10 kg in the first year, 7.6 kg over two years, and 6.1 kg at the five-year follow-up. 34% of participants who initially achieved remission of T2D continuing to remain in remission, underlining the program’s long-term effectiveness in metabolic health improvement [18–20]. Sustained weight loss was closely associated with improvements in overall metabolic health such as better control of hypertension, diabetes management, and triglyceride levels [18]. It should be noted that at 24 months, 17 (11%) intervention participants as compared with only three (2%) control participants had weight loss of at least 15 kg [19]. A notable limitation of the DiRECT study includes its lack of ethnic diversity, as the racial and ethnic characteristics were typical of populations in Scotland and Tyneside, UK, generally white European, and may not be generalisable to other groups such as South Asians, who tend to develop diabetes with less weight gain. This restriction may impact the applicability of findings to diverse global populations [18]. Patient acceptance and adherence was also low with only a 35% uptake of the trial and a significant dropout rate of 32% [18, 19].

The Cambridge IWMP has shown remarkable results, particularly in severely obese individuals aiming to either avoid or optimise for bariatric surgery. In a study conducted in the UK, between 2009 and 2013, participants adhering to a structured, multi-disciplinary approach across three 8-week phases, experienced substantial weight reductions, with women and men losing an average of 18.64 kg and 22.46 kg respectively, reducing the overall need for bariatric surgery by about 50%. Furthermore, about 69% of participants achieved a weight loss of at least 10% [21].

The Doctor Referral of Overweight People to Low Energy total diet replacement Treatment (DROPLET) trial provides further evidence for the efficacy of structured interventions using the IWMP Cambridge Weight Plan. This randomized controlled trial (RCT), conducted in the UK, included an 8-week TDR phase, followed by a 4-week food reintroduction and ongoing behavioural support for weight management. At the 12-month evaluation, 73% of participants had been reassessed, revealing that those in the TDR group experienced significantly greater weight loss, averaging 10.7 kg, compared to 3.1 kg in the usual care group. The adjusted mean difference stood at -7.2 kg (95% CI -9.4 to -4.9 kg). Moreover, 45% of the TDR group lost at least 10% of their initial body weight, as opposed to 15% in the usual care group. This weight loss was sustained over three years, with the TDR group maintaining an average loss of 6.3 kg compared to 2.7 kg in the usual care group. Significant improvements in diastolic blood pressure were observed, although other cardiovascular and metabolic markers showed no significant differences from the control group. A limitation of the DROPLET study was its initial design to assess outcomes at only one year, with a subsequent extension to three years being a post-hoc decision, which may influence the interpretation of long-term data as the study was not originally powered or designed to assess outcomes beyond the first year [6, 22]. The study reported a 30% dropout rate at 12 m of the intervention [22].

A retrospective study, which took place in Ireland, examined a Milk-Based Meal Replacement Programme for patients with severe obesity, starting with an 8-week TDR phase followed by 16 weeks of weight stabilization and maintenance. Out of 78 participants, initial weight dropped significantly from 144 ± 26 kg to 121.2 ± 24 kg, but some weight was regained by the third and fourth years, stabilizing at a moderate net loss of 4.7 kg to 7.0 kg from baseline. Weight regain was inversely related to initial weight loss and participant age, and directly related to the follow-up duration. The study’s retrospective design and the variable duration of follow-up among participants pose limitations, potentially introducing bias and affecting the generalisability of the results [23, 24].

Continuing the exploration of the impact of IWMPs, The DiRECT-Aus study, conducted in primary care settings across New South Wales, Australia, evaluated the efficacy of a 13-week TDR phase using Optifast products in participants with newly diagnosed T2D. This was followed by 8 weeks of structured food reintroduction and 31 weeks of supported weight maintenance. The study, involving 155 participants, reported an average adjusted weight loss of 8.1% (95% CI 7.2–9.1) after 12 months, with 56% achieving T2D remission. However, the absence of a control group, the open-label design, and the singular focus on a specific dietary intervention without a comparative analysis may compromise the robustness of the findings, limiting their applicability to diverse populations [25].

Similarly, The DIADEM-I trial demonstrated the effectiveness of intensive lifestyle interventions for young adults with early-stage T2D in the Middle East and North Africa. This RCT compared an IWMP, which included a TDR phase followed by gradual food reintroduction and physical activity support, against usual medical care [26]. Over 12 months, the intervention group exhibited a significant average weight loss of 11.98 kg compared to 3.98 kg in the control group, with an adjusted mean difference of -6.08 kg. Additionally, 21% of the intervention participants lost more than 15% of their body weight, markedly higher than the 1% in the control group. Diabetes remission was achieved by 61% of the intervention group, significantly surpassing the 12% in the control group. However, the trial’s open-label design and the focus on a specific regional population may limit the generalizability of the findings to other global populations. The interventions were also delivered by a specially trained team, which may not be available in typical clinical settings, potentially limiting the practical implementation of the program in broader healthcare contexts [27].

The STANDby trial sought to evaluate the effectiveness of a structured weight management program, specifically designed for South Asians with T2D, through a TDR phase followed by food reintroduction. Conducted among South Asians in the UK, the RCT utilized a randomized control design and an expanded observational cohort to assess both the immediate and delayed effects of the intervention. The results were promising, demonstrating that TDR led to significant weight loss and T2D remission comparable to outcomes observed in primarily white populations in other studies. The intervention group, comprising 13 participants, achieved an average weight loss of 7.7% and saw T2D remission in 5 individuals, versus a 1.2% weight loss with no remissions in the 12-person control group (*p* = 0.005). However, the study was not without limitations. The sample size was relatively small, and the study’s findings are limited by its single ethnic focus, potentially affecting the generalisability of the results to all South Asian populations. Moreover, the trial faced significant disruptions due to the COVID-19 pandemic, which impacted participant follow-up and data collection, possibly affecting the study’s outcomes [28].

### GLP-1RA

The efficacy of GLP-1 RAs for weight loss and remission of type 2 diabetes in the general population of obese adults is well documented and recommended by the UK’s National Institute for Health Care and Excellence (NICE) when used as a part of a specialised weight management service [29, 30].

In the Semaglutide Treatment Effect in People with obesity (STEP) clinical trials, which was conducted at 129 sites in 16 countries in Asia, Europe, North America, and South America, semaglutide 2.4 mg has demonstrated substantial efficacy in promoting weight loss across diverse populations [31–36]. In STEP 1, 1961 participants achieved an average 14.9% reduction in body weight over 68 weeks, substantially outperforming the placebo group’s 2.4% reduction, with 86.4% of the treatment group losing at least 5% of their body weight [31]. When used as a standalone treatment, this effect diminished in a two-year extension of STEP 1, stabilizing at a 5.6% reduction [32]. STEP 3 underscored the value of intensive behavioral therapy, with a 16.0% weight reduction at 68 weeks significantly outpacing the 5.7% seen with placebo [34]. In the 68-week STEP 4 trial, participants who continued semaglutide treatment for the entire duration, experienced significant weight loss, totaling 17.4%, whereas those switched to placebo after the initial 20 weeks regained weight, ultimately achieving a net weight loss of only 5.0% [35]. Lastly, STEP 5 demonstrated the durability of weight loss with semaglutide, maintaining a 12.6% point reduction over placebo at two years [36]. These results are promising for the potential use of semaglutide in broader applications. Its consistent safety profile and potential benefits for conditions associated with PCOS are promising, with gastrointestinal effects being the primary adverse events noted [31–33].

However, unlike IWMPs, GLP1-RAs have been trialled specifically in women with PCOS and reported to induce weight loss and improve markers of metabolic morbidity [37]. A recent systematic review, encompassing studies published up to July 2022, identified eight trials evaluating the efficacy of GLP-1RAs in women with PCOS. Descriptive analyses from this review revealed that GLP-1RAs were superior to placebo in improving anthropometric outcomes. Meta-analysis revealed no significant differences between exenatide and metformin in terms of anthropometric, biochemical hyperandrogenism, or metabolic outcomes, except that metformin resulted in a greater reduction in fasting blood glucose compared to exenatide [37]. More recent studies demonstrate the efficacy of semaglutide in obese women with PCOS, providing insights into both immediate and sustained weight management. In a 2023 study, 27 women with PCOS unresponsive to lifestyle changes alone were treated with semaglutide (0.5 mg subcutaneously, once weekly) for six months. The treatment resulted in a mean weight loss of 11.5 kg and improvements in metabolic parameters. Additionally, 80% of participants experienced normalization of their menstrual cycles, highlighting the potential efficacy of semaglutide in managing PCOS-related symptoms [38]. Furthermore, another study published in 2024, examined the long-term outcomes in 25 obese women with PCOS who continued metformin treatment for 2 years after a 16-week intervention with Semaglutide. During semaglutide treatment, significant weight loss occurred, decreasing from 101 kg to 92 kg, and metabolic and endocrine parameters improved. Two years post-semaglutide withdrawal, approximately one-third of the weight loss was regained, rebounding to 95 kg, but 84% of participants maintained a lower body weight than at baseline (*p* = 0.003). While cardiometabolic improvements reverted toward baseline, reductions in free testosterone levels persisted, indicating some lasting benefits of the intervention [39].

## Discussion

This review has identified many studies which demonstrate the substantial efficacy of IWMPs and GLP-1 RAs in treating and managing individuals living with obesity and metabolic disorders across varied demographic and clinical settings, and suggestions of some promising outcomes from GLP-1RAs for women with PCOS. However, despite an inherently higher risk of metabolic morbidity, there is a notable absence of research focused on the application of IWMPs specifically for women with PCOS.

Studies of both IWMPs and GLP-1 RAs report an average weight loss of 5% for up to 5 years following cessation of treatment. Weight regain is seen as a major hurdle to sustainability of most interventions and research highlights the value of concomitant behavioural treatment for successful weight maintenance. The health benefits of weight loss programmes are reported to last for up to 5 years after the end of the programme [40]. A weight loss of 5% is associated with multiple metabolic and cardiovascular risk factor benefits with a greater weight loss of 11–16% increasing these benefits further. However, it may be necessary to achieve at least a 16% weight loss or more for improvements in conditions such as non-alcoholic fatty liver and symptoms of obstructive sleep apnoea [41].

Although GLP-1 RAs have been trialed in women with PCOS and report encouraging outcomes, the quantity of published data is limited and the quality of evidence is low, with future research being recommended [37]. Women with PCOS demonstrate intrinsic insulin resistance independent of age and body mass index, exacerbated by obesity [42, 43]. Up to 75% of lean PCOS women and as high as 95% of obese PCOS women are reported to have insulin resistance [43]. Insulin resistance results in hyperinsulinemia which further increases androgen levels to amplify this risk [1]. Metabolic morbidity worsens in individuals who are overweight and obese with a linear correlation to adiposity [44, 45]. As women with PCOS demonstrate a significantly higher prevalence of overweight, obesity and central obesity, the risk of metabolic morbidity is exacerbated [46]. Long term weight gain in women with PCOS is also higher than those without PCOS [47]. The risk is higher in women from some ethnic backgrounds, especially those from South Asia [48], is likely to present at an earlier age as compared to women without PCOS [49] and in populations with increased socioeconomic deprivation [50].

Current interventions recommended for women with PCOS are not effective in reducing metabolic morbidity in this group. Lifestyle advice, which is universal, is delivered in primary care, and includes advice on an active lifestyle, exercise and diet. This has shown minimal improvement in weight loss in the short and long term [51]. Insulin sensitisers such as metformin improve insulin sensitivity but have minimal impact on weight loss [52–54]. They are variably used off label and often discontinued due to side-effects [55]. Intensive regimes for exercise are moderately effective in inducing short term weight loss but are not sustainable in the long term [56, 57]. Bariatric surgery is suitable for only a small subset of women with a BMI > 40 kg/m^2^ [58]. The incorporation of behavioural therapy into any weight management strategy has shown promise [59].

Amongst other factors discussed earlier, adequate uptake of interventions remains a significant barrier to treatment effectiveness. Currently there is insufficient patient involvement in creation of service models that would enable adequate uptake of interventions and matching of service models to patient needs [15]. Other factors include patient acceptability and adherence sustainability, effectiveness and side effects [16, 17].

Despite the promising potential of IWMPs and GLP-1 RAs in managing metabolic morbidity in women with PCOS, several limitations must be acknowledged. A critical issue is the lack of PCOS-specific RCTs evaluating IWMPs, making it difficult to draw definitive conclusions about their efficacy in this population. Additionally, existing studies often lack long-term follow-up data, raising concerns about the sustainability of weight loss and metabolic benefits over time. Furthermore, there is limited ethnic and socioeconomic diversity in current research cohorts, which may limit the generalizability of findings. The variability in intervention protocols across studies complicates direct comparisons, reducing the ability to establish standardized clinical guidelines. Another notable gap is the insufficient engagement of patients in the design and implementation of weight management programs, which may impact adherence and long-term success. Recent evidence suggests that mito-nuclear communication plays a crucial role in PCOS pathophysiology, with mitochondrial epigenetic modifications affecting nuclear gene expression and metabolic regulation. Studies indicate that epigenetic changes in mitochondrial DNA (mtDNA) and nuclear-related mitochondrial genes can influence metabolic dysfunction in PCOS, reinforcing the need for targeted therapeutic strategies to address these molecular pathways [60].

To address these gaps, future research should prioritize the design and execution of large-scale, long-term RCTs specifically targeting PCOS populations. Integrating behavioral therapy alongside IWMPs and GLP-1 RAs could improve adherence and maximize treatment efficacy. Furthermore, efforts should be made to enhance study diversity by including participants from various ethnic and socioeconomic backgrounds, ensuring findings are broadly applicable. Personalized treatment approaches that consider metabolic, reproductive, and psychological factors may also improve outcomes for individuals with PCOS. In addition, novel interventions beyond IWMPs and GLP-1 RAs should be explored, including alternative pharmacological agents and lifestyle-based therapies tailored to PCOS-specific metabolic challenges. Further investigation into the mechanistic pathways of GLP-1 RAs, including their impact on mito-nuclear communication and mitochondrial epigenetics, could provide a deeper understanding of their role in PCOS treatment. Therapeutic strategies such as time-restricted eating, which has been linked to improvements in mitochondrial function through epigenetic modifications, may hold promise as adjunct treatments for metabolic dysfunction in PCOS patients.

## Conclusion

Polycystic ovary syndrome is associated with an increased risk of metabolic morbidity, particularly in the presence of obesity. This review highlights the potential role of interventions such as IWMPs and GLP-1 RAs in managing these risks but acknowledges the limited robust evidence specific to women with PCOS. While these interventions have demonstrated benefits for weight loss and metabolic improvement, challenges remain, including variability in patient uptake, adherence, and long-term sustainability. Additionally, factors such as socio-economic deprivation, ethnicity, and intrinsic insulin resistance further emphasize the need for targeted research.

To address these disparities, future clinical trials should focus on tailored approaches that incorporate behavioral therapy, patient-centered service models, and culturally sensitive interventions. Expanding research on effective long-term strategies is essential to better understand and mitigate the metabolic and cardiovascular risks associated with PCOS, ultimately improving health outcomes and reducing inequities in affected populations.

## Key References

- Teede HJ, Tay CT, Laven JJE, Dokras A, Moran LJ, Piltonen TT, et al. Recommendations From the 2023 International Evidence-based Guideline for the Assessment and Management of Polycystic Ovary Syndrome. J Clin Endocrinol Metab. 2023;108:2447–69. 10.1210/clinem/dgad463.
-
  - This guideline provides comprehensive, evidence-based recommendations for PCOS management, addressing gaps in care and research priorities with globally relevant strategies for improving metabolic, reproductive, and psychological health.
- Goldberg A, Graca S, Liu J, Rao V, Witchel SF, Pena A, et al. Anti-obesity pharmacological agents for polycystic ovary syndrome: A systematic review and meta-analysis to inform the 2023 international evidence-based guideline. Obes Rev. 2024;25:e13704. 10.1111/obr.13704.
-
  - This systematic review evaluates the effectiveness of anti-obesity agents for hormonal, metabolic, and psychological outcomes in PCOS, highlighting limitations in existing research and paving the way for future pharmacological studies.
- Xie M, Yang Y, Zhang J. The effects of behavioral intervention on anthropometric, clinical, and biochemical parameters in patients with polycystic ovary syndrome: a systematic review and meta-analysis. Front Endocrinol (Lausanne). 2024;15:1297841. 10.3389/fendo.2024.1297841.
-
  - This meta-analysis demonstrates that behavioral interventions significantly improve weight loss, BMI, waist circumference, and depression in women with PCOS, but emphasizes the need for longer-term studies to determine sustained outcomes.

## Data Availability

No datasets were generated or analysed during the current study.
